# A Unified Model of Resilience and Aging: Applications to COVID-19

**DOI:** 10.3389/fpubh.2022.865459

**Published:** 2022-05-24

**Authors:** Andrew Wister, Katarzyna Klasa, Igor Linkov

**Affiliations:** ^1^Gerontology Research Centre, Simon Fraser University, Vancouver, BC, Canada; ^2^University of Michigan School of Public Health, Ann Arbor, MI, United States; ^3^United States Army Corps of Engineers, Engineering Research and Development Center, Vicksburg, MS, United States; ^4^Carnegie Mellon University, Pittsburg, PA, United States

**Keywords:** resilience, aging, systems, unified model, COVID-19

## Abstract

Drawing on multidisciplinary research focusing on a spectrum ranging from individual experience to structural system-level risk response and resilience, this article develops a rationale for a Unified Model of Resilience and Aging (UMRA). In response to a broad range of adversities associated with aging, it details the ways in which some individuals are able to bounce back better than others, or adapt better than expected, termed resilience. However, resilience and aging theoretical models have developed out of different disciplinary developments, ranging from individual levels to structural level complex systems, including several gerontological theoretical models addressing adaptation to life course and aging processes. The article reviews and synthesizes prior conceptual and theoretical work, and their empirical groundings, in order to develop an integrated resilience model with wide applications to aging-related problems including chronic illness, mental health, widowhood, poverty, caregiving burden, etc. The article focuses specifically on COVID-19 pandemic risk, response and resilience in order to specify applications of the UMRA, and to suggest avenues for future research and testing of theoretical axioms.

## Background

Over recent decades, research into elucidating what constitutes successful and healthy aging has increasingly recognized the importance of resilience as foundational in theoretical and empirical modeling (1, 2). Ungar (3) broadly defines resilience as a dynamic adaptive process through which individual traits, characteristics of their environment, and their internal and external resources are utilized in the face of adversity. Others have conceptualized resilience as the ability to bounce back from adversity or to cope better than expected compared to others based on harnessing physical, social and psychological resources over the life course (4). In the context of disasters, the National Academies of Sciences (NAS) has defined resilience as “the ability to plan and prepare for, absorb, recover from, and adapt to adverse events” (5). Similarly, the World Health Organization (WHO) applies the concept of resilience to health, defining it in terms of processes and skills that result in good individual and community health outcomes in the face of adverse threats, events, and hazards, which they have integrated as a priority area (6). More recently, health policymakers and researchers have begun to focus on systems-level resilience (7). For example, health systems resilience—defined as “the ability to prepare for, manage (absorb, adapt and transform) and learn from shocks”—is becoming a policy priority in order to deal with growing catastrophic, often cascading, shocks (i.e., pandemics, climate change, economic crises) (8, 9). Core elements embedded within the various conceptualizations of resilience include: (1) exposure to risk or adversity, (2) positive adaptation (3) individual variations, (4) protective factors that promote positive adaptation, (5) a dynamic process that requires longitudinal data, and (6) multidimensionality (10). Despite sharing common theoretical bases, these definitions demonstrate the diverse ways that resilience is conceptualized–from the individual to the social system, across various adversity contexts, and through different disciplinary lenses.

Adversity can take a variety of forms as people age, encompassing decline in physical and mental health, widowhood, poverty, homelessness, caregiving burden, social isolation, etc. The recent COVID-19 pandemic has underscored the well-established fact that older individuals who are more vulnerable in terms of socio-economic deprivation, living situation, social isolation, as well as health condition, are at higher risk of adversity than others. For instance, older people living in the community or long-term care (LTC) with common pre-existing physical conditions (e.g., respiratory diseases, cardiovascular disease, cancer, diabetes, obesity, and in particular multimorbidity), and those with pre-existing mental conditions, are at significantly increased pandemic risk sequalae on COVID-19 viral risk, morbidity and mortality; psychological well-being; depression, distress and anxiety; and social isolation (11–15). Formative research is accumulating evidence that some individuals facing ‘pandemic adversity' can cope and recover better than others and has linked this to resilience processes (16–20). For instance, a recent COVID-19 study found that, while 93 per cent of a sample of older adults reported vulnerabilities directly linked to the pandemic, approximately two-thirds identified positive responses to this adversity—what they concluded represented a form of resilience (17).

Therefore, resilience applications to gerontology are required, given the unique age-related challenges that shape vulnerability, risk, and resilience, such as normal aging physical and cognitive declines (brain health), increased likelihood of multimorbidity, and other health-related problems. This approach draws attention to salutogenic (healthful) processes connected to quality of life and well-being; and furthermore, it balances the ongoing emphasis on pathogenic (psychopathology and disease) processes (21–23). A resilience perspective encourages the exploration of positive pathways, coping thresholds and adaptive protective processes, multiple types of resources, and interventions that foster resilience (24). Yet, there is an absence of both a single measure and a unified model of resilience to guide research in part because of the focus on different adversity contexts, disciplinary variations in foci, and the fact that resilience approaches to aging are relatively nascent.

The purpose of this article is to: (1) review theoretical and empirical developments in the field of resilience and aging; (2) develop a unified model of resilience and aging that integrates emerging knowledge in this field; and (3) apply this model to COVID-19 response and adaptation. Research into how and why some individuals can (or cannot) respond positively to adversity has far reaching implications for understanding adaptation and informing future healthy aging policies.

## Review of Resilience Models and Evidence

### Early Developments

Resilience research originated with individual-based applications to children and adolescents drawn from developmental psychology (25, 26). These initial explorations of resilience focused on psychological outcomes, such as mental health and mental illness, and adjustment to trauma (25–28). The resilience qualities that researchers pinpointed were termed protective factors or resources, such as self-efficacy, mastery, or parental/social support (28–30). The identification of these resilience components helped to identify the potential of malleable dimensions of social-psychological resilience. However, it does not elucidate their interconnections, clarify how individuals access or use these resources to overcome adversity, how the macro-meso-micro environments link, or how life course trajectories may affect the accumulation or erosion of resilience (26, 31, 32).

Conceptually, resilience processes and measures were viewed as an evolution of stress theory, in which adaptation and coping are the product of stressors interacting with risk and protective factors (33–35). The application of stress theory moved beyond the individual and led to an ecosystemic perspective that acknowledges the inter-dependence between social and environmental system levels (30, 36, 37). This afforded an opportunity for resilience research to transition into a new phase in which multi-level processes were recognized as important in the adaptation process (36, 38–40).

A subsequent wave of resilience research applied knowledge from the first several decades of research to develop effective interventions aimed at enhancing well-being, function, and preventing psychopathology among different populations, including gerontology (4, 25, 27, 28). However, applied research has been hampered by underdeveloped theoretical/conceptual models of resilience—measures of resilience typically focused on the individual, often utilizing social-psychological measures—and underlying problems with conceptual complexity. More recently, resilience models have been applied to a wide spectrum of adversities affecting older adults, which has led researchers into diverse conceptual landscapes that traverse the micro-meso-macro environment (28, 41). Some examples include: family/interpersonal resilience and aging (42, 43); genetic, brain health and physical resilience (28, 44, 45); multimorbidity resilience (38, 46–48); resilience and mental health (1, 28, 41); successful aging (1, 26, 28, 45); work, retirement and resilience (49); cultural specific resilience (38, 49–51); and system-level disaster resilience affecting older populations (52–54). Furthermore, in a comprehensive review of 77 studies using growth mixture modeling to examine forms of resilience among adults experiencing different adversities, Infurna & Luthar (40) conclude that there is a need to consider different methodologies given the complexity of resilience; identifying processes leading to resilience; and the necessity for a multidimensional approach. Still, there remains an absence of a unified multi-level model of resilience and aging that can be applied to different adversity types experienced within and across individual and societal locations, and which can guide resilience research into the next developmental phase.

### Theoretical Building Blocks Adapted From Gerontology

Resilience and aging has its roots in a family of social-psychological, sociological, and socio-environmental concepts and gerontological theoretical models addressing adaptation to life course and aging processes and stressors (27–29, 41). We identify the most relevant axioms for resilience based on dominant models, beginning with applications to the individual, followed by socio-ecological and system-level models.

At the individual level, foundational work in developmental psychology conceptualizes optimal development as a dynamic balance between gains and losses that result in ‘successful aging' (24, 31, 55–59). The classic stress-coping (SC) model (33–35) has been used to understand adaptation to adversity articulating the moderating or buffering effects of social support in the stress process to reinstate homeostasis. The model of assimilative and accommodative coping (AAC) comprises two forms of adaptation to cognitive appraisals of adversity: modification of life circumstances to assimilate or reinstate balance; and adjustment of life goals to accommodate incongruence (31, 56, 60, 61). Similarly, positive adaptation to aging has been explained using three interlocking processes embedded in the widely employed selection, optimization and compensation (SOC) model (55). Selection refers to our choice of focal life areas; optimization is the access and application of appropriate resources; and compensation is the enlistment of alternate means to maintain function (56). The SC, AAC and SOC models suggest that stress buffering and positive adaptation (a primary component of resilience) is maximized when older individuals align desired goals with the resources that they have at their disposal (55). For instance, Wiles and colleagues (51) found in their study of disability challenges that the most resilient older adults were those who were able to maintain high value activities of daily living even if facing the negative effects of multimorbidity (61).

Many gerontological models consider adaptation as the absence of adversities, such as the successful aging paradigm (58, 59), or creating a state of homeostasis between stressors and adaptive responses (33–35, 37). Resilience models, on the other hand, consider a wider spectrum of positive responses that may protect, reinstate wellness, and even promote growth (41, 62). A cluster of models within the sub-field of positive psychology and applied to aging shift attention from ‘coping' with adversity to more favorable forms of adaptation and rehabilitation. Positive psychology can be defined as the pursuit of the adaptive, creative, and emotionally fulfilling aspects of human behavior (24, 63). It is fostered by the strengths and resources (i.e., individual resilience) of people, founded on a deeper understanding of the salutogenesis of health, which moves our focus from pathology to healthy adaptation and promotion of well-being (4, 21, 63, 64).

A major gap in the individual-level psychological models and theories that form major precursors to resilience approaches is that they do not fully explain the processes underlying how the individual is interconnected to multi-level domains constituting their environment, including the physical infrastructure in which individuals live, policy arenas, their social and community networks, and access to information (40). Information resilience entails the availability and harnessing of information needed in decision-making embedded within complex systems, including the competence and literacy level required for it to be acted upon. In this article, we develop a resilience framework for research, practice, and policy that serves as a bridge between individual and structural dynamics.

Socio-ecological and complex systems models help fill this gap. Socio-ecological (or socio-environmental) (SE) theory posits that individuals, social systems, and the environment are interrelated and interdependent (37, 65). Applications of the SE perspective to aging have been prolific, focusing on a range of environmental areas, including housing (66), homelessness (67), green spaces and walkability (68), and healthy public policy (37, 69). This body of theory is useful in resilience research in that it underscores not only the importance of environmental domains to positive adaptation, but also the concept of an optimal zone of development and/or adaptation, and environmental resources that can be embedded in these domains.

At the structural level, a complex systems approach to resilience (e.g., in the case of natural disasters and pandemics) attempts to link and model the different individual and environmental-level networks discussed within existing SE frameworks (51, 52). According to the National Academy of Sciences (NAS), the ability of a system to plan, absorb, recover and adapt in response to adversity represent four key resilience processes (5). Connelly and colleagues (70) argue that, across diverse socio-ecological application domains, resilience features in common include critical functions (services), thresholds, cross-scale (both space and time) interactions, memory, and adaptive management. Multiple social and ecological determinants of health (such as poverty, societal perceptions of race, educational opportunities, and the home or institutional physical environment) vary across physical, social, cognitive and information resilience domains and can influence the health outcomes of aging or older individuals. Interconnectivity of these four environmental resilience domains (physical, information, cognitive and social) and key resilience structural processes (plan, absorb, recover and adapt) constitute a system model wherein overall resilience can emerge based on systemic properties and interactions (53). The application of a complex systems approach to resilience has the potential to knit together macro-meso-micro factors, all of which need to be integrated in an overarching model of resilience and aging. However, systems models typically overlook the individual resilience elements and processes due to a focus on structural dynamics of resilience.

Finally, given the recent application of resilience models to aging, the temporal dimension can be understood within lifecourse theory, which places an aging lens to the dynamic interplay of structural (i.e., historical, institutional, community and cohort-related) and individual (i.e., social resources and agency) factors (71–73). Lifecourse theory articulates how lives can be shaped by period or historical circumstances; how intra-cohort variation in exposure and adaptation to adversity occurs; and how earlier life experiences can either deteriorate resilience (cumulative disadvantage) or inoculate a person from the adversities of later life (learned responses to chronic illness) (33, 74). Indeed, life course theory has developed into a primary bridging theory (and can be synthesized with other theories) within aging studies and gerontology that connects the individual and structural domains within a dynamic understanding of human development and aging (75). For instance, O'Rand and Hamil-Luker (76) found that early childhood socio-economic and environmental disadvantages increase the risk of cardiovascular disease in old age. On the other hand, coping ability may be enhanced when human agency is learned and reinforced over time. In this sense, lessons learned from one experience of adversity may enable the development of coping skills needed for subsequent recovery. A “resilience trajectory” is therefore the accumulation of previous lifecourse experiences and resources, coupled with non-mutable genetic and partially mutable personality and social factors (4, 26, 36, 77). Table 1 lists and describes foundational and current theories and models, contributions to resilience and aging thinking, level of analysis, and limitations.

**Table 1:** Foundational gerontology theories and resilience and aging models.

**Model**	**Contribution to aging resilience**	**Level of analysis**	**Limitation(s)**
Developmental Psychology Models • Stress-Coping • Assimilative & Accommodative Coping • Selection, Optimization, & Compensation	Stress-buffering and positive adaptation maximized when older individuals adapt to stressors, and/or align goals with available resources	Individual-level	Does not fully explain resilience processes; limited elucidation of how individual is interconnected to external multi-level domains; aging-related limitations such as chronic illness deemed as a ‘not successful aging'. Some models do not address unique aging contexts
Successful Aging Model	Adaptation conceptualized as the absence of adversities where individuals strive to create a state of homeostasis between stressors and adaptive responses		
Positive Psychology Models	Shift attention from “coping” with adversity toward adaptation and rehabilitation		
Life Course Theory	Lives are shaped by period/historical circumstances and early life experiences can lead to variation in exposure and adaptation to adversity (provides a temporal perspective to resilience)	Individual-level, Cohort-level, or Structural level	Primarily used as a bridging theory(individual and structure) in gerontology; not directly linked to resilience concepts
Socio-ecological / Socio-environmental Model	Individuals, social systems, and environmental are interrelated and interdependent, underscoring importance of environmental domains to positive adaptation (i.e. optimal zone of development and/or adaptation in each domain)	Individual-level nested within Environmental-level Domains	Does not directly connect multi-level domains (macro-meso-micro factors) to resilience concepts; focus is on ecological domains
Metatheory of Resilience and Resiliency	Identification of resilience processes; individual's biopsychospiritual homeostasis state, disruption requires adaptation, role of resources, leads to reintegration and growth	Individual-level, embedded in social networks	Absence of an aging focus; concentrates primarily on individual experiences; does not include structural and system-level domains
Developmental Psychological Models of Resilience	Individual's, developmental processes central to overcome adversity experiences in early life, role of crises	Individual, Social-psychological	Focuses primarily on childhood and developmental adaptation, omits structural and system level; peripheral aging context
Comprehensive/Integrated Models of Resilience	Bioecological systems framing generalized resilience; initial attempts at integration primarily starting with individual focus	Genetic, Individual, Social-psychological, and System-level	Lack unique aging application and contexts; resilience processes assumed
Formative Resilience and Aging Models (e.g., Nested Models of Resilience)	Individual and socio-ecological approaches to resilience and aging; nested models of influence	Individual-level, Social-psychological, and System-level	Focus either on individual or ecological domains; lack elaboration of resilience processes; uneven conceptual and operationalizations of resilience
Life Course Model of Multimorbidity Resilience	Builds on Metatheory of Resilience and Resiliency through specific applications to multimorbidity and aging	Individual-level and Social-level nested within Socio-ecological Domains	Social-psychological framing dominates model; developed specifically for multimorbidity and aging
National Academies of Sciences Disaster Resilience Model	Links different individual and environmental-level networks in socio-ecological models into resilience processes (plan, absorb, recover, adapt)	Nested Complex Systems-level	Does not include aging and lifespan development; focus on structural levels
Resilience Matrix	Combines the National Academies of Sciences system functions (plan/prepare, absorb, recover, adapt) with system domains (physical, information, cognitive, social), aligning with the socio-ecological model	Nested Complex Systems-level	Does not include a life course perspective; limited aging contexts
Integrated Systems Model of Resilience	A bioecological systems framing of resilience that emphasizes dynamic, temporal, and multisystem pathways, as well as cascading effects	Nested Complex Systems-level	Focus and application limited to children's development

## Gaps and Potential Integration of Resilience and Aging Models

Resilience models range in terms of applications to different types of adversity, different life stages and populations, disciplinary approaches, and in terms of a focus on either individual psychological resilience or on the structural or environmental system (28). Furthermore, resilience model development specific to aging has been hampered by a preoccupation with a variety of issues, including (a) whether resilience is a trait or a process, and their components; (b) whether it entails a protective effect from adversity, recovery/reintegration, and/or growth; (c) whether aging itself is an adversity to which resilience can be applied; (d) what constitutes adversity and how to measure it; and (e) how to methodologically operationalize and analyze resilience, including utilization of qualitative, quantitative or multi-method approaches (1, 27, 41, 62). Nevertheless, there are important theoretical developments in resilience and aging. We review a selection of common resilience models, conceptualizations, and issues to identify: (1) gaps and limitations; and (2) where a unified model for aging and resilience could fill gaps in knowledge through integration (see Table 1).

There are numerous resilience models that aim at understanding individual-level coping at the psychological level. Most resilience research has incorporated individual psychological or social-psychological concepts/models, such as psychopathology, positive psychology, self- efficacy, self-esteem and control, adaptation to stress, coping, or successful aging (28, 41, 62, 78, 79). While psychological models have elaborated on the factors and processes that affect resilience, including the recognition that some resources foster resilience (e.g., social support) and that the adversity or trauma itself can fall outside of the individual; they have primarily focused on individual experiences. Furthermore, the conceptualization of resilience has largely been framed using established theories/models related to processes of adaptation or coping to aging generally (see above) rather than one specific to resilience. Moreover, at times, they simply provide a definition of resilience without an established model. In cases in which a model has been generated, it has tended to apply only to a particular type of adversity or subpopulation, in part, to simplify the model and align with research knowledge in a sub-field.

The Metatheory of Resilience and Resiliency introduced by Richardson (27) proposes that life stressors create adversity that disrupt an individual's biopsychospiritual balance or homeostasis state, unless protective factors are invoked. The outcomes of resilience reintegration (growth, full, partial, or dysfunctional reintegration) are the consequence of coping mechanisms and skills driven by what is called “resilience energy,” originating from the individual, but also from external sources. The Lifecourse Model of Multimorbidity Resilience (LMMR) extends Richard's metatheory, applied specifically to multimorbidity adversity among older adults (4). The resilience process moves clockwise from adverse life events to the final process of wellness-recovery/growth, including activation of resources embedded in the individual, social and environment, in support of coping processes (4). The LMMR contends that successful activation of social resilience entails harnessing available resources embedded in the individual, social and environmental spheres; however, a social-psychological framing dominates the model.

More recently, models of resilience have expanded the focus outward to encompass socio-ecological and/or systems approaches (29, 36, 39, 52–54, 80). However, similar issues have persisted with respect to conceptualization, application to aging, and individual-structural integration. Drawing from the SE framework, early work by Wild and colleagues (39) created a model of six nested domains to reflect contextual and collective dimensions of resilience for persons in later life: individual, household, family and neighborhood, community, and society. This model helps one visualize the interconnectedness and interdependence of multiple life spaces in relationship to resilience. However, what is still lacking is a model that connects not only the individual to structural types, but also the resource domains and processes. A more comprehensive systems approach to resilience has been offered by Masten (36), in which a bioecological systems framing generalized resilience emphasizes the dynamic, temporal and multisystem pathways and cascading effects. Masten (36) states that, “from a systems approach, resilience refers to the capacity for successful adaptation to disturbances that threaten system function, viability, or development” (p. 298). This model raises important issues and insight into the importance of levels of analyses, use of longitudinal data, and moreover, the need for integrated science to address new challenges in resilience research across levels, including poverty, family conflict, disaster, disease epidemics, and global climate change. While Masten's Integrated Systems Model of Resilience identifies the promise of a bioecological approach, the focus and application is on children's development.

Other applications of systems models of resilience have been applied to gerontology, including ones originating from disaster research (53, 80). A Resilience Matrix has been developed (80, 81) that combines a SE model with the National Academies of Sciences (NAS) system functions and the Network-Centric Warfare domains into four stages of resilient systems and applies these to healthy aging. The major resilience processes include system-level ability to respond to adversity through (a) planning/preparation; (b) absorption; (c) recovery; and d) adaptation, which occur within and across within physical, information, cognitive and social domains. But, the challenge of applying a systems lens to a full set of aging contexts which balance a spectrum of spheres of influence and response, ranging from the individual to the larger organizational nested systems, still remains.

## A Unified Model of Resilience and Aging

**Figure 1:**
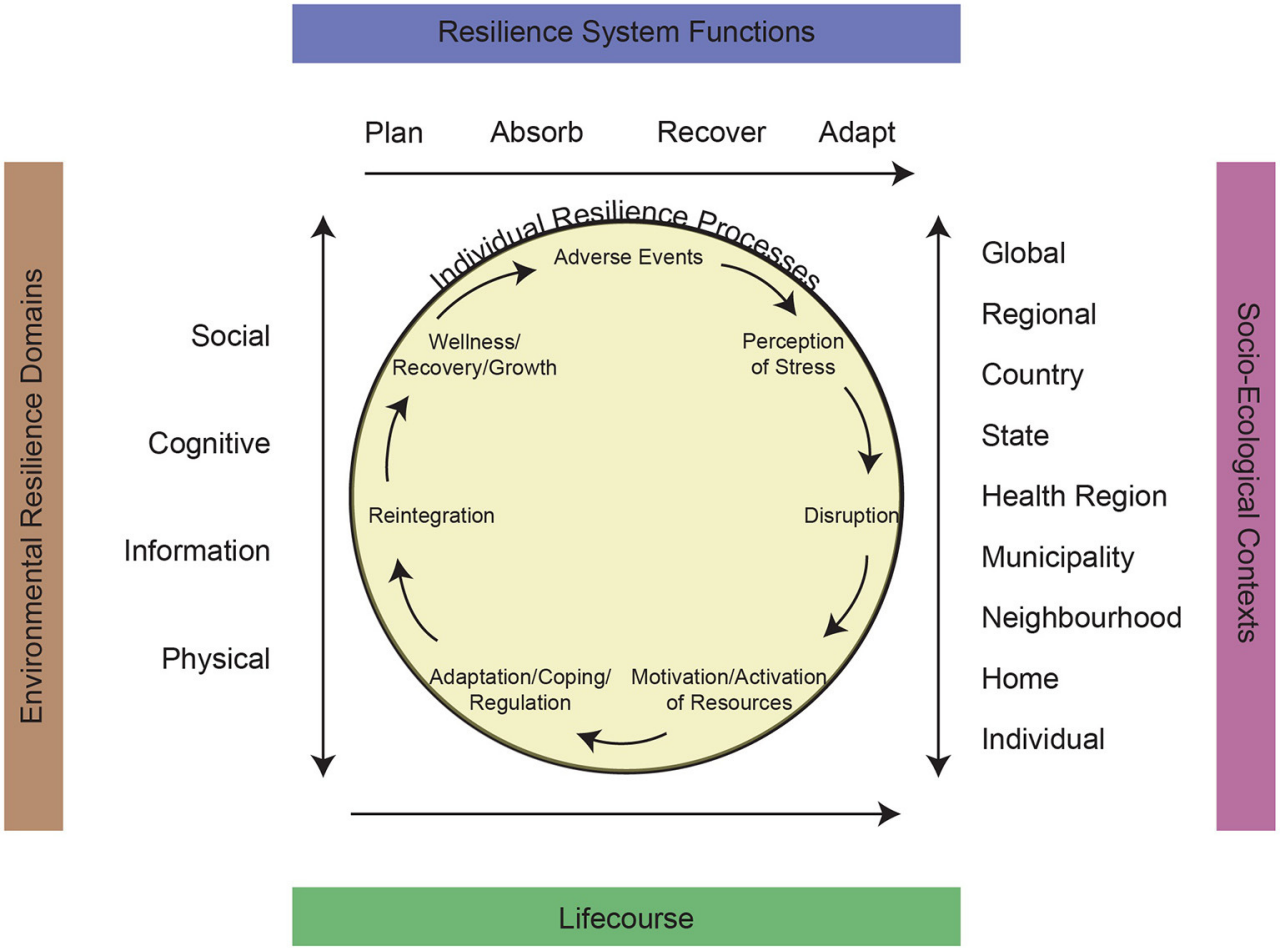
A unified model of resilience and aging.

Based on developments in the field of resilience and aging, a Unified Model of Resilience and Aging (UMRA) needs to integrate: processes that operate at the level of the individual and those that affect the environment, including: (a) social contexts; (b) individual lifecourse; (c) domains of system resilience; and (d) functionality of resilience in the system (see Figure 1). The UMRA assumes a complex set of adversities, resources, and processes that occur over the lifecourse of the individual to promote resilience.

### Individual Resilience Processes

We begin with the core of the UMRA wherein individual strength-based processes can be identified as part of a dynamic adversity-wellness wheel (Figure 1). Richardson (27) describes a biopsychospiritual model in which resilient qualities are harnessed in order to respond to stress-induced disruption. Similarly, we start with the presence of an adverse event, which works through stress perceptions to create a level of disruption that can affect the health and well-being of the individual. Furthermore, it upholds the value that disruption (adversity) is necessary for growth and to access latent human potential (27).

The following phase of the process is the internal or external activation of resources, which require motivation, energy, and access (27, 77). Internal activation of resources is an expression of agency. External activation of resources includes support from a friend or family member, or from cultural capital, identity, or coping strategies. Resource mobilization can entail a combination of individual, family, social or environmental factors. Risk and vulnerability factors can interfere, complicate, and even delay positive resilient outcomes (79). Risk factors include a range of known epidemiological influences (e.g., demographic, social, environmental, cultural, lifestyle, and health and behavioral social determinants), some of which are mutable (physical activity), and some of which are not (genetics). Additionally, as risk and vulnerability factors increase in their influence, the ability to rely on individual resources decreases, and social/environmental support becomes more important (3).

The effective activation of resources engages a set of protective processes of coping and emotional regulation, which support reintegration of a sense of self, social roles, and positive adaptation. Examples of coping include assimilative and accommodative processes, as well as selection, optimisation, and compensation embedded in the SOC Model (55). As the resources and processes work synergistically to effect resilience, the individual progresses toward wellness, recovery, and potential growth. The resiliency model proposes four levels of reintegration that may occur following a disruption to homeostasis. The uppermost outcome, resilient reintegration, entails growth, knowledge, self-understanding, and importantly, increased strength of resilience resources (4, 23, 27). Alternatively, individuals may reintegrate back to a state of homeostasis, which is characterized by recovery, healing, and overcoming a negative event. Reintegration with loss or dysfunctional reintegration refers to individuals who either deny or simply accept their condition.

### Socio-Ecological Contexts

Resilience experienced at an individual level must be understood within the broader socio-ecological landscape in which individuals and systems interact. The right-hand side of the UMRA figure lists several interlocking social and environmental domains drawn from SE theory (37, 82). While not exhaustive, domains include the individual, family, neighborhood, municipality, health region, state, country, regional and global. These nested contexts are relevant for understanding both the origin and trajectories of risks and vulnerabilities and the targeting or system level responses to respond to adversity. The SE model contends that older people are embedded in family, community, organizational and socio-political environments that ultimately affect their aging processes (37, 82). The SE model illustrates the interconnections of these domains through interlocking spheres of influence from the individual level to the global level. SE domains encompass a spectrum of systems including institutional factors, community factors, public policy, intrapersonal factors, and interpersonal processes (52). Furthermore, it is consistent with a complex systems model of resilience because it provides a framework for understanding risks and their disruptions to various nested spheres of influence and their relationship to individuals.

### Environmental Resilience Domains

The resilience literature applied to aging and older adults has focused on several types of resilience and their interactions. Based on our review of literature, and building on the author's prior work, our proposed UMRA include four domains that we term environmental resilience: social, cognitive/psychological, information, and physical (41, 59, 82, 83). These represent broad resilience categories, and additional sub-types of resilience can be identified that fall within these overarching forms, such as cultural resilience, genetic resilience, etc. We define these generic types as follows: (1) *Social resilience* can be understood as the maintenance of positive social interaction, including community participation or social engagement. Conversely, significant levels of social isolation can result in negative adaptation to adversity in old age. (2) *Cognitive resilience* pertains to the ability to cope with stressors created by adversity. For example, an individual needs the ability to establish an understanding of their baseline health and needs, but mental health conditions or dementia could hinder any behavioral change needed to adapt to adverse events. (3) *Information resilience* recognizes the importance of literacy, knowledge, and access to information resources that promote understanding of resilience pathways and solutions to adverse events. For example, information resilience can refer to the individual's competence and the literacy level, as well as access to information, required to make resilient decisions within the environment that they reside in. (4) *Physical resilience* relates to the interconnection between the person and their environment at the genetic, physiological and functional levels reflecting one's ability to complete tasks of daily living, social roles, and remain functionally active within their environment. The resilience domains are understood to have unique and interacting effects on adversity outcomes.

### Critical System Resilience Functions

We incorporate the NAS core resilience functions: plan, absorb, recover, and adapt. These largely draw from research conducted on natural disaster response and other forms of environmental adversity, but have been more recently applied to adversity linked to aging (52–54). Each of these critical functions are central to positive resilience responses to adversity conditions. First, planning and preparing for adverse events requires targeted reductions in risk and vulnerabilities, in response to an identified threat. The greater the understanding of the characteristics of the form of adversity, such as population susceptibility, severity, and pathways, the greater the avenues for planning. Absorption of stressors and outcomes associated with an adversity is necessary in order to sustain critical functions and initiate a resilience through recovery and adaptation. Since resilience entails supportive elements resulting from life course experiences and underlying strengths, the degree to which an individual or system can absorb adversity is central to a positive or negative response. Recovery takes shape through the various forms of strength-based resilience types. Some recovery is short-term and others long-term, each of which has different implications for aging outcomes such as healthy aging. Recovery is essential to counteract the weakening of any system. Limitations or shortcomings inevitably exist. Thus, resilience provides another process to safeguard critical functions over time.

The resilience functions are broad-based by design with the intention of incorporainge multiple levels of analysis from the individual to the societal. Specific critical functions may differ in terms of their relative importance to ensuring a positive resilience response depending on the level of analysis. These critical functions attempt to balance between *learned resilience* and *innate resilience*. For example, an individual may or may not need to plan to be resilient due to factors that allow greater resilience to be achieved at birth or early life experiences that remain throughout the lifespan. Similarly, some individuals can plan to overcome difficulties but still have less resilience than others with less planning. These individuals have their own unique level of *innate resilience* (an individual sliding scale of their own minimum and maximum level of resilience). The resilience functions can help an individual and a population enhance their *learned resilience*, which allows them to reach maximum levels of resilience, increasing their own positive resilience response to a significant degree (small or largel).

Overall, resilience as a component of a multi-layered system must be understood in the context of risk and adversity interconnections. Risk associated with various adversities is connected to the size, seriousness, and length of a potential threat, and interacts with the vulnerability or susceptibility of a complex system. The preparation, absorption, recovery and adaptation critical functions work synergistically to define resilience thresholds and to identify potentials for prevention and restoration. By incorporating a critical systems framing, our model attempts to encompass and apply to all levels of the socio-ecological framework, from the individual to the broader community and environment. While for some specific individuals planning and preparing may not matter to their individual level of resilience, a focus on preparedness and planning at the societal level can lead to improved population health outcomes among older individuals, more robust aging in place policies, and a stronger safety net that can help maximize how many individuals can have increased positive resilience outcomes.

### Lifecourse Dimension

The temporal/historical dimension of the model recognizes that aging is time-dependent and that individuals form lifecourse trajectories against the backdrop of social change. Lifecourse theory provides a linkage between structural (i.e., historical, institutional, community and cohort-related) and individual (i.e., social resources and agency) factors that influence health and social trajectories of individuals as they age (71–73, 75, 77, 82). Human development entails lifelong processes that are influenced by the timing and intensity of early life experiences, events and transitions. Early life trauma may weaken resilience in the short-term or provide experiential learning to strengthen resilience in the long-term. Second, individuals proactively employ human agency, which is a tenant of resilience modeling (41, 80). Third, historical events influence experiences and trajectories, such as the proliferation of fast food production and consumption, its effect on obesity, and subsequent effect on diabetes (83). Fourth, life course emphasizes that lives are interconnected, and shaped by our social networks, especially close family (74). Finally, lifecourse risks and resources (e.g., genetics, literacy, knowledge, wealth, health, social relations, identity, competence, etc.) create opportunities (advantages) or adverse conditions (disadvantages) that influence how life stressors are experienced (74, 77, 84). In their review, Rybarczyk and colleagues (74) show how accumulated life experiences can inoculate older persons to negative health conditions. Together, life course dimensions indicate a potential “resilience trajectory” that is dynamic and embedded in temporal aging processes.

## Applying the UMRA to the Coronavirus Pandemic

### Coronavirus Disease (COVID-19) Pandemic Adversity

COVID-19 is a newly discovered form of the SARS virus that is highly contagious and linked to adverse morbidity and mortality outcomes (15, 85–87). The appearance of COVID-19 in early 2020 and its rapid spread into a pandemic has forced some of the most dramatic social, political, and economic transformations and adaptations observed for many decades. Given the pronounced deleterious consequences that it has had on older populations, the current pandemic is particularly relevant as a case study for theoretical applications of the UMRA (80).

COVID-19 has claimed millions of lives and infected hundreds of millions of people worldwide. Moreover, these figures likely underestimate its prevalence because of limited testing capabilities and resources. It is noteworthy that between 30 % and 40 % of positive cases are among persons 60 years of age and older; and over 80 % of deaths are among this group, with the lions share occurring in long term care (LTC) facilities (15, 87). Thus, it is not surprising that the current COVID-19 crisis is viewed as a “gero-pandemic” that begs an aging lens (69). This requires consideration of the actual and perceived risk, seriousness, vulnerability, and individual to societal reactions to the pandemic (12). As such, a resilience and aging model should provide a deeper understanding of these pandemic tensions.

The next sections apply the UMRA to specific pandemic risk, response, and resilience based on accumulated research. We examine the UMRA socio-ecological contexts and system-level critical functions across the resilience domains, and connect these to the individual-level resilience processes of disruption due to pandemic adversity, resource activation, adaptation to stressors, reintegration, and restoration of well-being.

### Socio-Ecological Context of UMRA and COVID-19

There are a significant number of research initiatives that have focused on policy contexts affecting the COVID-19 pandemic, especially within hospital or long-term care environments; however, only a small sub-set of studies have specifically applied a socio-ecological model and include resilience considerations. In a study of COVID-19, frailty and long-term care, Andrew et al. (88) utilize a socio-ecological framework to understand pandemic vulnerabilities linked to a series of nested contexts ranging from individual to system-levels. At the individual level, the high level of physical and cognitive impairment and frailty, coupled with higher rates of pre-existing conditions that predispose residents to higher levels of COVID-19 infection and deleterious outcomes is at the core of risk, response, and resilience. At the family/friendship network level, on the one hand, risk of infection increases when visitation is allowed without extreme protective practices, but on the other hand, physical separation exacerbates feelings of social isolation and loneliness among residents (88). Some technological and informational solutions (e.g., smart phone or computer video media used to connect family/friends with residents), mitigated social isolation. Additionally, cyber approaches to support complex systems in fostering resilience during disasters has been shown to be necessary to react to intense periods of adversity (54).

At the institutional level, facility group programs, resident councils, and congregate meals also increase potential spread of COVID-19, even though these same programs are central to maintaining and maximizing resident quality of life. Facilities that were able to react quickly to circumvent these programs reduced infection rates once COVID-19 had entered the facility (88, 89). In addition, spread of COVID-19 has been linked to other institutional organizational and policy contexts, including testing ability, availability of personal protective equipment, resident and congregate room size, staff training in infectious disease, and synchronized administrative organization for mitigation strategies, all of which can be highly variable across health care jurisdictions and facilities. Similarly, at the community level, the safety procedures of the public transportation system and the level of infection in the catchment area of the facility, especially if within a marginalized community, affects risk levels (88, 90).

The policy arena reveals some of the most striking challenges during the pandemic. The availability of resources and funding, such as within health area, state and national levels is at the root of many of these issues. Health catchments with greater resources are able to utilize disease surveillance systems, testing, personal protective equipment, and infectious disease experts, as well as infusing a range of other supports. Laxton and colleagues (89) utilize a socio-ecological framework to identify key policy areas requiring attention in order to strengthen pandemic response, such as: collaboration across health care sectors; federal direction and collaboration with other government levels in pandemic response policy development; reforming the LTC regulatory system to improve disease contagion mitigation; and reducing systemic inequalities among population sub-groups facing increased risk of disease and lower access to services. The interconnections of these socio-ecological contexts in terms of risk, response, and resilience, helps in understanding the ways in which they create positive and deleterious synergies.

### The Individual Context of UMRA and COVID-19

At the individual level, initial COVID-19 research has offered evidence that resilience is critical for coping with and navigating a variety of pandemic adversities (91–93). For instance, Grossman et al. (91) showed a moderating effect of resilience between COVID-19-related loneliness and sleep problems among older adults. Another study reported that low and normal resilience groups of older adults experienced increases in mental distress compared to a high resilience group (92). Two additional studies found that older persons who engaged in proactive coping at the start of the pandemic were able to reduce the level of pandemic stress and maximize psychological well-being over the first wave of the pandemic (18, 19).

Moreover, there are many other risk factors that could impact how resilient an individual was during the pandemic. For example, prolonged isolation due to stringent quarantine policies, lack of hobbies or extracurricular activities, or political unrest and limited ability for civic engagement could have affected an individual's social resilience. Additionally, a lack of healthcare access—specifically geriatric and mental health care—or experiencing an adverse health event such as a stroke, a fall, relapse in substance use, or Alzheimer's disease can influence brain/cognitive resilience. Specific to the emerging field of brain resilience, prior research has identified two processes of brain reserve applied to Alzheimer's Disease (AD) (94), that can help to understand individual resilience to pandemic stressors, such as meeting functional needs, especially when support systems were broken during peak infection periods. (1) *Passive reserve* suggests that there is a threshold of brain capacity that may affect one's ability to deal with the adversities created by the pandemic for person's with significant cognitive decline. (2) *Active reserve* pertains to individual coping processes (pre-existing or compensatory processes, such as using memory training techniques) that allows individual to maintain function (94), which may be more important in affecting the ability of an individual with cognitive decline (such as AD) to recover and adapt to pandemic stressors at an individual level. The latter active reserve process is particularly important within an integrative resilience framework, since it provides a theoretical account for why individual-level forms of resilience, even at the brain level, are embedded in the other domains (94, 95).

Next, information resilience could have been hindered by fake news campaigns, poor public health communication, inability to read or write in a country's official language, and dementia. Last, lack of mobility, prior or newly onset mental health or physical disability, loss of housing, and even a loss of home health can degrade an individual's physical resilience. The UMRA offers insight into the processes by which individuals harness resources embedded in the socio-ecological environment to build and sustain resilience to protect against and recover from pandemic adversity.

### Resilience System Functions for Pandemic Response in the UMRA

The NAS system-level critical functions include plan, absorb, recover, and adapt, all of which are embedded within the socio-ecological domains discussed above and directly influence the five resilience domains (52, 80). The integration of complex system critical functions has been underscored during the pandemic. This has been particularly accented among vulnerable older adults, such as those living in LTC, marginalized groups with limited access to resources, and those with pre-existing conditions, to name a few (15, 52, 80).

The system-level critical functions in the UMRA provide specification and direction for individuals, localities, and nations to respond to disasters such as the COVID-19 pandemic. While we present these separately, there are important feedback loops that allow each critical function to inform the others in a fluid manner. The *preparation* and *planning* element is crucial to reducing vulnerabilities prior to the appearance of an adverse condition or set of conditions. At a policy level, this can take many forms such as access and application of high-level data that can be used evaluate areas of vulnerability to a threat (54, 95). Detailed epidemiological population health data on pre-existing conditions, living environments of older populations, environmental scans of organizational preparedness (especially LTC) and their available resources, and multidisciplinary pandemic response team availability, are examples of potential areas. At an individual and family level, fostering psychological resilience to protect against the stressors associated with a pandemic and mitigation (e.g., physical distancing, lockdown, etc.) comprises a core preparation function (11, 95). Risk perceptions form another important behavioral barrier or facilitator to protective health (96). In addition, computer literacy and access to advanced technology can contribute to planning and preparedness levels, providing the ability to respond quickly to COVID-19 (54, 97).

The second function is the ability of individuals, communities, and societies to *absorb* pandemic stressors. The highest mortality rates during the pandemic thus far have occurred among older adults living in long-term care (LTC) (including congregate living environments, retirement homes, supportive housing, assisted living, etc.). Older persons living in congregate settings tend to have physical and/or cognitive challenges, and are often treated as complex patients, placing them at the lowest levels of resilience. This points to the need to strengthen the resilience of surrounding systems within which they live (98, 99). Living in group quarters with group-based activities; congregate meals; high levels of frailty and cognitive impairment; and having more severe and complex pre-existing conditions increase disease risk and deleterious outcomes, especially among the most marginalized (race, ethnicity, social-economic status, sexual orientation) as vulnerabilities and system-level environmental conditions tend to be weaker (100). Financial resources of a facility can result in staff being required to re-use personal protective equipment because of shortages, lower staff training levels, and low staff-resident ratios for disease prevention and protocol implementation.

Examples of how differing levels of pandemic stress can be absorbed across resilience domains are evidenced in the experiences within the LTC systems within communities. For instance, ground zero for the COVID-19 outbreak in the US occurred in Seattle WA, where the disease found a foothold that allowed its high level of contagiousness to be realized. In response, the University of Washington Medicine's (UWM's) Post-Acute Care (PAC) Network rapidly developed a three-phase coordinated approach (101). In facilities with low numbers of cases (phase one) the focus was on communicating response plans; implementing disease tracking methodologies; and distribution of personal protective equipment. In phase two facilities with rising caseloads, emphasis was placed on pandemic education and training of staff and administration; and surveillance and testing. The third phase was directed at facilities with rapid spread. The UWM integrated multi-disciplinary “drop-teams,” comprised of MDs, RNs and disease specialists, that were sent to direct high-level disease response, including full range testing for COVID-19; triage and transfer of patients if needed; and coordination with local public health agencies (101).

The ability of systems to *recover* represents a third critical function of the UMRA. The long-term care and community care systems in most countries have revealed serious vulnerabilities affecting recovery during the COVID-19 pandemic. Mitigation approaches at the ground level (e.g., ramped up testing and isolation; staggering and/or eliminating congregate meals and programs), coupled with policy and regulatory changes (e.g., reducing care aides and nurses working at multiple sites; disallowing family/friendship contact from outside of the facility) helped to shift LTC systems toward recovery (89, 101). However, full recovery requires successful vaccine roll-out and compliance, given that many of the system-level issues are systemic problems.

Policies focusing on the community-level environment need to adopt innovative approaches to maintain independent living in the right place with adequate supports to meet daily needs, such as food, medication, necessary health care, and safe methods of social contact. A combination of translating lessons learned, new programs and service models, and capacity building across systems will be needed to find the path to pandemic recovery. At the individual level, new norms of behavior (e.g., mask wearing, vaccine passports, improved testing) will be required to recover and shift into the final adaptation phase.

*Adaptation* is the final phase of system response to the COVID-19 pandemic; however, iterative processes of system change occur along all critical function trajectories. Successful system-level adaptation to the pandemic will require careful assessment and evaluation of efficacy and effectiveness of programs and practices, significant investment of resources, and organizational and legislative reform (89, 98). This phase will directly feed back into the planning phase to prepare for the next pandemic or crisis.

#### UMRA Lifecourse Dimension and COVID-19

The lifecourse of individuals is fluid and occurs within the context of history, aging, and social structure (75). Some studies have shown that older adults adapted better to lockdown and social/physical distancing mitigation policies better than younger adults, suggesting that they are able to employ learned lifecourse experiences of coping to adversity to the pandemic (12). Alternatively, there may have been traumatic life experiences earlier in life that have eroded resilience capacity. Lifecourse theory also emphasizes that people have linked lives. Burke (102) found that older adults with intergenerational support systems adapted better than those without such buffers during the pandemic. Individual experiences must also be understood within the larger systems and institutional structures in society. As reported above, older adults in LTC facilities with better pandemic organizational policies and procedures created more resilient environments for their residents (88, 89). Additionally, the axiom of human agency was shown to be critical during the pandemic as evidenced by the influence of advocacy and social movements that identified vulnerable/marginalized groups during the pandemic and the need to tailor interventions to their needs (103, 104). Finally, the lifecourse dimension is fundamental to understand resilience from an aging lens, given the primacy it places on the fluid and temporal nature of the aging processes.

## Research Gaps and Opportunities

There are several research gaps and areas of opportunity that may improve model enhancement, application, and the development of interventions aimed at heathy aging. First, the measurement of resilience remains controversial with no agreed upon operationalization, index or scale (79, 105). Indeed, the measurement of resilience has been highly diverse, in part due to it being anchored in a diverse number of conceptual frames, including psychological, emotional, spiritual, physical/functional, economic, cultural, social and ecological resilience (4, 28, 41, 49, 75, 105, 106). A number of measurement approaches have been used. These entail, for instance, estimating “buffering” effects of hypothesized protective factors in the effect modification, scale construction, comparison of resilience characteristics between predefined groups, data-driven subgroup identification in the latent class analyses, assessing predictors of adversity-outcome residual values in regression analyses, and stressor-response patterns in high-density time-series based on a systems approach (41, 75). One of the most measures is the Connor-Davidson Resilience Scale (there are several versions), measuring the degree to which individuals perceive that they can overcome stress and adversity in life through a general set of questions (107). It shares similarities with other resilience measures, such as the Brief Resilient Coping Scale (108), and the Family Resilience Scale (109), in that a number of self-reported items are used to capture resilience (e.g., how well do you bounce back from a problem). Other measures have been developed to measure multimorbidity resilience among older adults based on a multi-domain (functional, social, psychological) index (46, 110). Albeit, there is yet to exist a quantitative or qualitative measure that fully captures an integrated model of resilience and aging, leaving this task to future research (105). Thus, new measures are needed to coincide with the new integrated theoretical/model developments that are pushing the frontiers of resilience thinking. Additionally, quantitative scales and indices require analysis of psychometric properties and testing using different populations (43, 105).

Second, mixed methods studies are needed to triangulate findings based on qualitative data pertaining to meanings and experiences of adaptation and with empirical quantitative data that capture individual and system-level processes (80). This will allow for a deeper understanding of the processes of resilience, especially as they filter down to the level of the individual in the socio-ecological system. The experiential component of resilience has to a large degree occurred in a knowledge arena separate from quantitative approaches.

Third, measurement of the domains encompassing the UMRA are needed to fully test its usefulness under differing adversity contexts. These include, for instance, health and health systems shocks (heat waves, flooding, fires, hurricanes), economic crises (2008 crash) and at the individual level such as unexpected health decline (cancer, hip fracture due to fall), loss of brain health (dementia), sudden homelessness, or death of a spouse/partner (80, 81). Fourth, applications of advanced modeling strategies will be needed to combine different levels of measurement and test assumptions of non-linear and reciprocal associations embedded in the model. Fifth, effective interventions need to be developed and tested based on the components of the UMRA, such as critical thresholds, teachable moments or periods of susceptibility to change in relation to differing levels of adversity, and limitations to resilience processes due to underlying genetic or other traits (29, 30). Sixth, multi-factorial interventions need to consider interactions among resources, including cascading influences (38), whereby improving a resource in one area strengthens another resource. Finally, interventions need to consider both intended and unintended consequences on a range of outcomes and over both short-, medium-, and long-term periods.

## Potential Practice-Oriented Applications

The application of this resilience model to the real world necessitates intermediate steps to develop program, policy and other knowledge translation approaches, and to evaluate and assess their merit. At a basic level, a resilience framework can help guide researchers, clinicians, and policy makers from different disciplinary and practice backgrounds in furthering their understanding of promising plans for informing interventions to overcome various types of life adversities (40, 80, 95). For clinicians or policy-makers to operationalize this model into their practice, a valuable initial step would be to differentiate resilience-by-design and resilience-by-intervention (81). Resilience-by-design assumes that a system can internally reconfigure to adjust and recover following a disruption, whereas resilience-by-intervention necessitates the development and application of external resources (95). The UMRA provide guidance to build internal resilience (resilience-by-design) in order to respond to future adversities (future pandemics or other environmental crises, mental or physical health challenges among older people, or long-term-care reform) by identifying and fortifying unique strengths and circumstances of an individual, community or system. For example, older people have experiential knowledge in dealing with life crises that can be leveraged for future adverse events. Additionally, the UMRA can be applied to developing and retrofitting external support systems (resilience-by-intervention) based on the identification of proven approaches. For instance, stockpiling resources that are needed during a crisis, or the application of relevant medications and treatments for combating mental health among older people (95).

## Conclusion

This article has contributed to the literature through a conceptual and theoretical review of resilience that bridges individual and structural system-level bodies of knowledge. By conducting multidisciplinary exploration and integration of research and theories related to resilience and aging, a Unified Model of Resilience and Aging (UMRA) is developed. Our model connects multiple sources of resources embedded in the individual, family, community, and society with a series of processes that occur during disruption and reintegration phases. It recognizes the non-linearity of the resilience process, and the potential for cascading crises that may restrict or delay resilient outcomes or for reversals, all of which are embedded in the dynamic lifecourse of individuals. The UMRA model elaborates the role and intersections of socio-ecological contexts and system-level critical functions across resilience domains and ultimately for individual processes of resilience experience. Based on available research and knowledge, the model is applied to the COVID-19 pandemic to reveal potential applications to one of the most severe and far-reaching forms of adversity experienced across the globe.

Continued research into the multidimensional, dynamic concept of resilience has the potential to uncover innovative ways to approach aging from a strength-based approach within differing contexts. It also helps to understand the well-being paradox, in that individuals facing challenges often redefine their well-being as a coping mechanism embedded in processes of resilience. Interventions require a deep understanding of how individuals, and the structural systems in which they are embedded, respond to internal and external threats to aging. The UMRA provides initial direction in identifying effective ways to address these issues. The remaining challenge is to test and further develop models of resilience and aging under unique adversity conditions, types, and across time and place.

## Author Contributions

AW primary lead role in development of the article. KK assisted with writing of all sections of the manuscript. IL assisted with conceptualization and writing of all sections of the manuscript. All authors agree to be accountable for the content of the work. All authors contributed to the article and approved the submitted version.

## Conflict of Interest

The authors declare that the research was conducted in the absence of any commercial or financial relationships that could be construed as a potential conflict of interest.

## Publisher's Note

All claims expressed in this article are solely those of the authors and do not necessarily represent those of their affiliated organizations, or those of the publisher, the editors and the reviewers. Any product that may be evaluated in this article, or claim that may be made by its manufacturer, is not guaranteed or endorsed by the publisher.
